# Author response to “Commentary on detoxification of deoxynivalenol by pathogen-inducible tau-class glutathione transferases from wheat” by Dr. Latika Shendre

**DOI:** 10.1016/j.jbc.2026.111139

**Published:** 2026-01-28

**Authors:** Herbert Michlmayr, Anastassios C. Papageorgiou

**Affiliations:** 1 Department of Agricultural Sciences, BOKU University, Institute of Microbial Genetics (IMiG), Tulln, Austria; 2 Turku Bioscience Centre, University of Turku and Åbo Akademi University, Turku, Finland

We sincerely thank Dr. Shendre for the encouraging comment on our work. We would like to emphasize that this was primarily a biochemical study demonstrating (for the first time) that tau-class plant glutathione transferases (GSTs) are in principle capable of catalyzing glutathione adduct formation with the Fusarium virulence factor deoxynivalenol (DON). As thoroughly discussed in the paper, the catalytic rates observed with DON were low, but we hypothesized that such GSTs may contribute to basal Fusarium resistance in plants. Whether this is true will indeed require further experimental verification.

Therefore, we fully share Dr. Shendre’s enthusiasm that future research should focus on the biological relevance of trichothecene-conjugating GSTs for Fusarium resistance and accordingly, also the metabolic fate of trichothecene-GSH adducts *in planta*. Given the high redundancy of plant GSTs (*e.g.*, 200 tau- and 87 phi-class GSTs known in wheat) with perhaps similar function, we doubt that knock-out of individual genes encoding GSTs such as “TaGST-02” or “TaGST-10” will cause a significant phenotype in wheat. However, we agree that constitutive overexpression of trichothecene-detoxifying genes is an adequate approach to study Fusarium resistance mechanisms of plants. As GSTs are highly versatile catalysts and their physiological roles in plants are still incompletely understood, such studies may also shed further light on the metabolic functions of GSTs, particularly in stress response.

Sincerely,

Herbert Michlmayr and Anastassios C. Papageorgiou (on behalf of all authors)

